# On Variolous Inoculation

**Published:** 1805-10-01

**Authors:** 


### *To the Editors of the Medical and Physical Journal.*

Gentlemen,

In your last Number, p. 144, Mr. Ring very properly remarks on the danger of suffering children to be carried abroad in London or its vicinity, previous to inoculation, on account of the number of persons loaded with small-pox, who, to the disgrace of the police, are suffered to be continually in the streets of the metropolis spreading disease and destruction. Unfortunately, keeping children within the house will not always preserve them from the danger, as these masses of contagion push their way into our very dwellings. A son of the Rev. Mr. C. of Tottenham Court Road, has nearly fallen a victim to the small-pox, propagated in this manner.

A shoe-maker in the neighbourhood having occasion to send some shoes to the house where this gentleman resides, employed a girl to carry them who was covered with the loathsome eruption. Mr. C's child, of three months old, being in the nurse's arms, passed this girl on the stairs. Fourteen days after, the eruption began to appear. The child was very ill and loaded with the confluent disease disease, but at length struggled through it with great difficulty.

Mrs. C. has suffered very severely from fever, pain, and inflammation of the breasts, occasioned by suckling the child, who communicated local pustules not only to her breasts but to her face and other parts of the body, to the amount of from forty to fifty.

When I sent my letter, which you have inserted in your last Number, page 140, I did not recollect the following deplorable and decisive instance of the mischief of variolous inoculation, published by Dr. Willan in his very instructive Reports on the Diseases of London. He therein says, p. 18, "I cannot here pass over a striking instance of the bad effects arising from partial inoculation. A child was inoculated in April 1796, whose parents kept a shop in a court, consisting of about twenty houses. As the inhabitants repaired every day for necessary articles to the source of infection, the consequence was, *that seventeen persons were affected with the small-pox in the natural way, within a fortnight after the child's recovery; and eight of them died of the disease.*"

It may not be amiss to call to the recollection of your Readers the following facts, asserted by Baron Dimsdale in his Thoughts on general and partial Inoculation.

Baron Dimsdale says, p. 9, "I received a letter from a poor man, who kept a school about eight miles from Hertford, to inform me, that not being able to pay a proper person, he had ventured to inoculate his own family himself, and begging a visit on account of one of his children who he feared was in danger. I complied with his request, and found one child dying of a confluent pock; but my compassion abated on finding his house filled with some poor neighbours, from whom he received a small gratuity for their inoculation, one of which had lost an eye under his care. This man's residence was in a small town, and from his patients, *several caught the small-pox, and some died.*

" I saw a poor woman dying of a confluent disease. Her husband had raised money for his own inoculation, and having had the disease favourably, was assured by a farmer, who inoculated him, that he might safely go home to his family. The wife died, leaving five children, who all had the disease and recovered.

" At a village not far from Hertford, the same farmer inoculated as many of the parish as could raise five shillings and three pence, informing the others that the smallpox was not catching from the inoculated; *but the whole neighbourhood became infected, and several died.*"

These instances tend to shew the necessity of legislative interference, if not altogether to prevent variolous inoculation, at least to confine it within some limits.

*August* 5, 1805. S. M.

